# Supplementary material for: Silencing lipid catabolism determines longevity in response to fasting
Source: Nat Commun. 2026 Jan 22;17:1919. doi: 10.1038/s41467-026-68764-y (PMC12923588; doi:10.1038/s41467-026-68764-y)
Supplement: Supplementary file 1 — Supplementary Information [file 41467_2026_68764_MOESM1_ESM.pdf]

Supplementary information:

Silencing lipid catabolism determines longevity in response to fasting

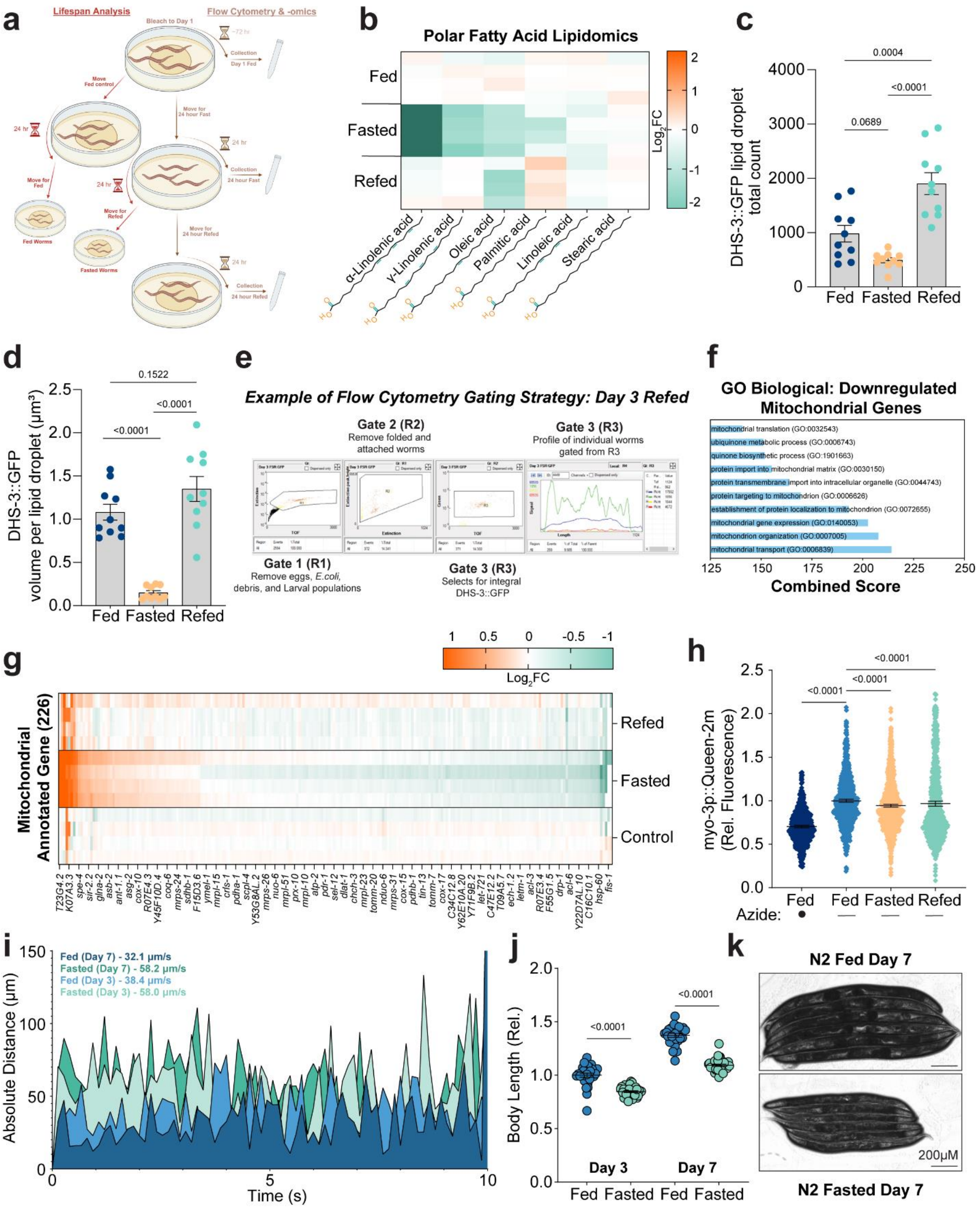

Supplementary figure 1: Fasting initiates lifelong physiological effects

a. Schematic of the fasting-refeeding experimental procedure. Created in BioRender. Tatge, L. (2026) https://BioRender.com/j3nf55o **b.** Heatmap depicts relative levels of long chain polar fatty

acids from *C. elegans*. Lipidomic analysis compares fed Day 1 adults to 24 hours of fasting and subsequent refeeding for 24 hours. n = 4. **c.** Quantification of total lipid droplet counts from DHS-3::GFP micrographs under fed, 24-hour fasting, and 24-hour refeeding conditions. n = 10, ordinary one-way ANOVA with Tukey's multiple comparisons test used for statistics. **d.** Quantification of volume per lipid droplet from DHS-3::GFP micrographs under fed, 24-hour fasting, and 24-hour refeeding conditions. n = 10, ordinary one-way ANOVA with Tukey's multiple comparisons test used for statistics. **e.** Gating criteria from the large-particle flow cytometry for Day 3 adults under refed conditions. **f**. Gene pathway analysis showing the transcriptional regulation of mitochondrial-annotated genes in *C. elegans* during fasting as Co-expression Predicted GO Biological Process 2018 combined scores. **g.** Heatmap depicts relative transcriptional changes in significantly differentially regulated mitochondrial-annotated genes. Analysis compares 24 hours of fasting and 24 hours of refeeding to fed adults. **h**. Scatter plot from large-particle flow cytometry of transgenic worms expressing the Queen-2m ATP sensor in body-wall muscle. Individual values ratio the fluorescent excitation at 405 nm to 488 nm with respective time-of-flight (TOF). Plot compares Day 1 adults under fed conditions to 24 hours of fasting and subsequent refeeding for 24 hours. Azide treatments serve as a negative control mitochondrial function by inhibiting Complex IV and blocking ATP synthesis. Mean ± 95% CI. n = 604, 922, 1217, and 843 from left to right, Kruskal-Wallis test and Dunn's multiple comparisons test used for statistics. **i**. Quantification of worm speed through automated video surveillance (Wormtracker) by measuring absolute distance covered over 10 seconds at Day 3 and Day 7 of adulthood under fed or fasted conditions. n= 5 worms per condition. **j**. Quantification of worm body length through automated video surveillance (Wormtracker). All values are made relative to the fed Day 3 adult worms. Mean ± SEM. n= 28, 37, 26, and 34 from left to right, ordinary one-way ANOVA with Šídák's multiple comparisons test used for statistics. **k**. Micrographs of Day 7 adult wild-type worms after fed or fasted at Day 1. Scale bar= 200µm.

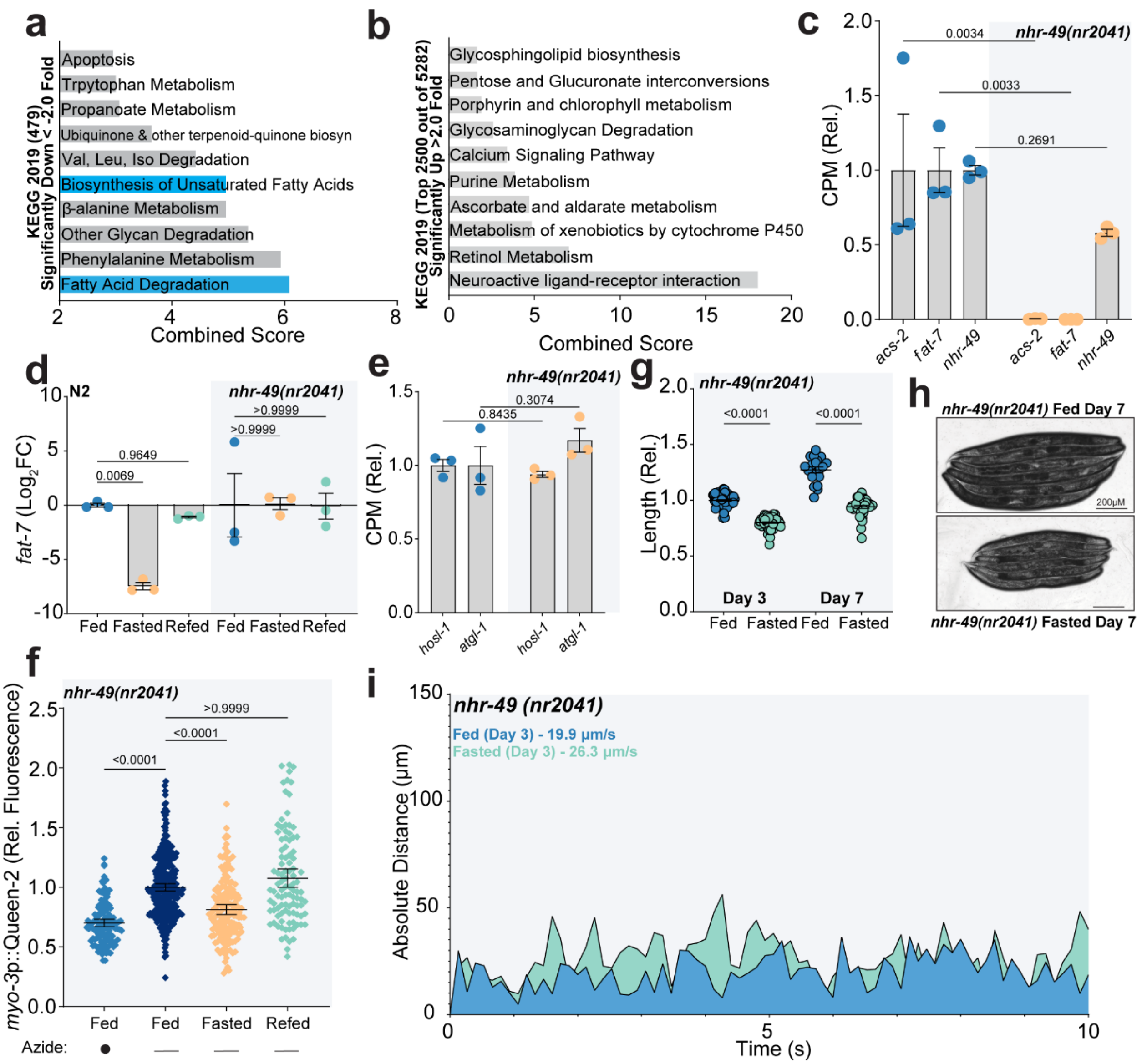

Supplementary figure 2: NHR-49 is dispensable for fasting-induced physiological benefits

a,b. Kyoto Encyclopedia of Genes and Genomes (KEGG) pathway analysis of genes differentially regulated during 24-hour fasting. Cut-off of 2.0-fold using combined score as a metric, (a) <-2.0-fold downregulated, (b) >2.0-fold upregulated with a cut-off of the top 2,500 genes out of 5,282. Highlighted in blue KEGG pathways relevant to lipid metabolism. **c.** Relative transcript abundance of the respective genes determined by RNA-sequencing. Shown are normalized reads in per counts per million (CPM) comparing N2 wild-type to *nhr-49(nr2041)* mutants. Mean ± SEM. n = 3, ordinary one-way ANOVA with Šídák's multiple comparisons test used for statistics. **d.** Relative abundance of *fat-7* transcription determined by reverse transcription quantitative PCR (RT-qPCR). Analysis compares wild-type (N2) and *nhr-49(nr2041)* mutant worms at Day 1 of adulthood under fed conditions to 24 hours of fasting and subsequent refeeding for 24 hours. Mean ± SEM. n = 3, ordinary one-way ANOVA and Tukey's multiple comparisons test used for statistics. **e.** Relative

transcript abundance of the respective genes determined by RNA-sequencing. Shown are normalized reads in counts per million (CPM) comparing N2 wild-type to *nhr-49(nr2041)* mutants. Mean ± SEM. n = 3, ordinary one-way ANOVA with Šídák's multiple comparisons test used for statistics. **f.** Scatter plot from large-particle flow cytometry of *nhr-49(nr2041)* mutant worms expressing the Queen-2m ATP sensor in body-wall muscle. Individual values ratio the fluorescent excitation at 405 nm to 488 nm with respective time-of-flight (TOF). Plot compares Day 1 adults under fed conditions to 24 hours of fasting and subsequent refeeding for 24 hours. Azide treatments serve as a negative control. Mean ± 95% CI. n = 152, 409, 215, and 100 from left to right, Kruskal-Wallis test and Dunn's multiple comparisons test used for statistics. **g.** Quantification of worm body length through automated video surveillance (Wormtracker). All values are made relative to the fed Day 3 adult *nhr-49(nr2041)* mutant worms. Mean ± SEM. n = 48, 57, 19, and 34 from left to right, ordinary one-way ANOVA with Šídák's multiple comparisons test used for statistics. **h.** Micrographs of day 7 adult *nhr-49(nr2041)* mutant worms after fed or subject to fasting as Day 1 adults. Scale bar= 200µm. **i.** Quantification of *nhr-49(nr2041)* mutant worm speed through automated video surveillance (Wormtracker) by measuring absolute distance covered over 10 seconds at Day 3 of adulthood under fed or fasted conditions. n= 5 worms per condition.

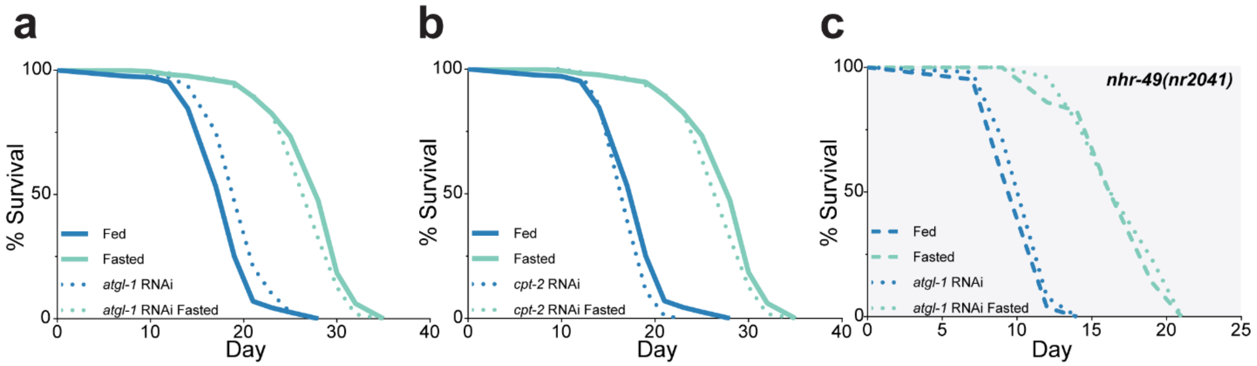

Supplementary figure 3: Upstream lipid catabolism genes are dispensable for fasting-induced lifespan extension

a.-c. Lifespan analysis of worms under fed or fasted conditions (24 hours of dietary deprivation at Day 1 of adulthood). Wild-type (N2) or *nhr-49(nr2041)* mutant worms were treated with (**a.**, **c.**) *atgl-1* RNAi or (**b**) *cpt-2* RNAi. Log-rank (Mantel-Cox) test used for statistics, see Supplemental Data Table 1.

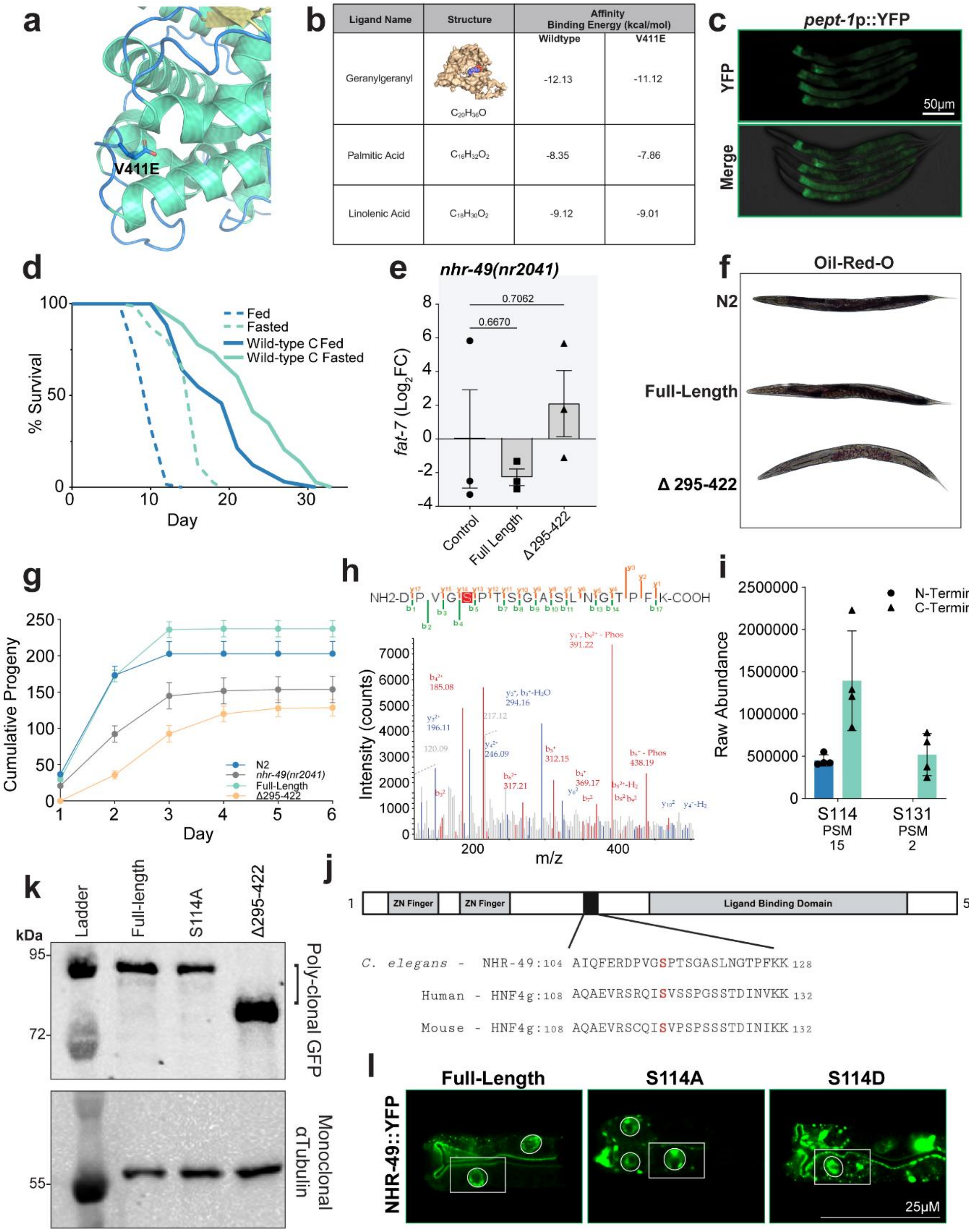

Supplementary figure 4: NHR-49 mediated activation and attenuation of lipid catabolism program in a ligand-independent fashion

a. Structural prediction depicts the gain-of-function V411E mutation within the base of ligand-binding pocket of NHR-49. β-sheets in yellow, α-helices in green and random coils in blue. **b.**

Predictive modeling utilizes AutoDock data of NHR-49 with and without the V411E gain-of-function mutation to model the binding of potential ligands. Created in BioRender. Douglas, P. (2026) https://BioRender.com/49d7k7w **c.** Fluorescence micrographs of transgenic *pept-1*p::YFP worms to confirm intestinal-specific expression from the respective promoter. This promoter was used as the original vector to clone NHR-49::YFP and all other mutations into. Scale bar = 50μm. **d.** Lifespan analysis of worms under fed or fasted conditions (24 hours of dietary deprivation at Day 1 of adulthood). *nhr-49(nr2041)* mutants or NHR-49::YFP ectopically expressed in the *nhr-49(nr2041)* mutant worms. Log-rank (Mantel-Cox) test used for statistics, see Supplemental Data Table 1. **e.** Relative abundance of *fat-7* transcription determined by RT-qPCR. Analysis compares *nhr-49(nr2041)* mutant worms ectopically expressing NHR-49::YFP full-length or Δ295-422 in the intestine. Mean ± SEM. n = 3 independent trials, ordinary one-way ANOVA with Tukey's multiple comparisons used for statistics. **f.** Micrographs of stained neutral fatty acids by Oil-Red-O staining in N2 (wild-type) worms ectopically expressing intestinal NHR-49::YFP full-length or Δ295-422 at Day 1 of adulthood. **g.** Brood count comparing eggs laid in N2 worms, *nhr-49(nr2041)* mutants, and mutant worms ectopically expressing intestinal NHR-49::YFP full-length or Δ295-422 worms over the first six days of egg-laying. n= 8 for N2, *nhr-49(nr2041)* and full-length worms. n=6 for Δ295-422 worms. **h.** Mass spectrum of the phosphorylated NHR-49 peptide at serine residue 114. **i.** Raw abundances of peptide fragments containing phosphorylated serine 114 or serine 131 from endogenous NHR-49, immunoprecipitated via biotin-streptavidin from N- and C-terminal TurboID-tagged NHR-49. n= 4 per group. **j.** Schematic depicts domain structure of NHR-49 and compares a polypeptide sequence within the hinge region with human and mouse HNF4γ. Highlighted are potential conserved phosphate conjugation sites. Created in BioRender. Douglas, P. (2026) https://BioRender.com/meuvlj3 **k.** Western blot analysis of NHR-49::YFP full-length, S114A, and Δ295-422 using polyclonal rabbit anti-GFP and monoclonal anti-tubulin for immunodetection. **l.** Fluorescent micrographs of transgenic worms in L3-L4 larval stages ectopically expressing NHR-49::YFP (full-length), the S114A mutation (S114A), and the S114D mutation (S114D). Scale bar= 25μm. n = 31, 35, and 64 from left to right over 2 independent trials.

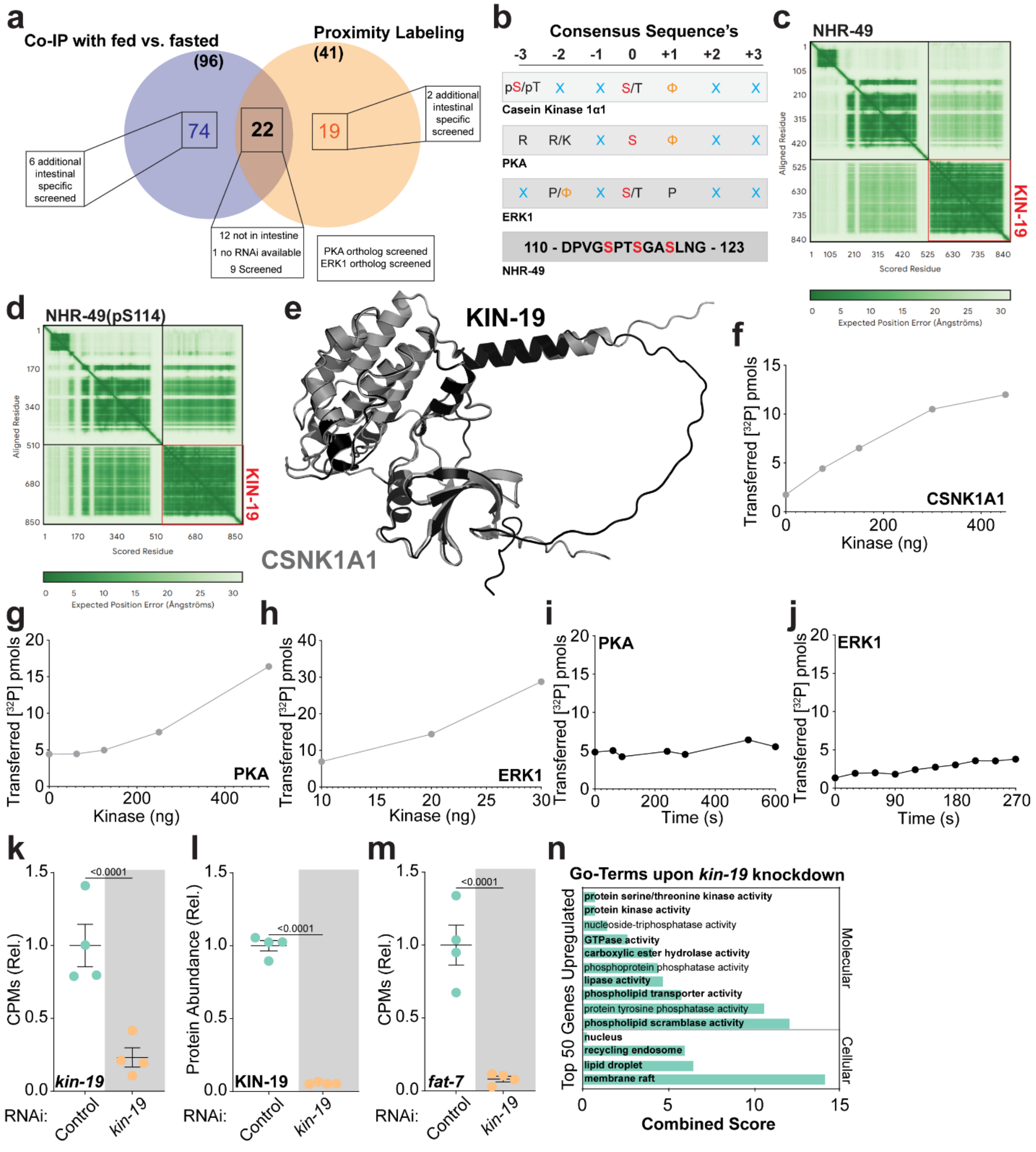

Supplementary figure 5: Priming event on S114 is necessary for KIN-19 phosphorylation

a. Venn diagram of two independent proteomics datasets showing kinases immunoprecipitating with NHR-49 constructs under varying conditions: fed versus fasted NHR-49::GFP, and endogenous TurboID-tagged strains. **b.** Schematic of consensus binding motifs of CSNK1a1, PKA, and ERK1 compared to NHR-49. **c,d.** Expected position error plot for interactions between NHR-49 and KIN-19 (**c**), phosphorylated S114 NHR-49 and KIN-19 (**d**) as determined by

AlphaFold3. See Fig. 3c. **e.** Predicted secondary structure of KIN-19 (black) and human CSNK1A1 (grey), modeled using AlphaFold2, an advanced AI-driven deep learning algorithm for protein structure. Alignment uses the surrounding amino acids of each respective active site (K130 to L143 on CSNK1A1 and K129 to L142 on KIN-19). RSMD = 0.036. **f-h.** Quantification of γ-phosphate transferred from [^{32}P] ATP to the wild-type NHR-49 polypeptide by recombinant (**f**) CSNK1A1, (**g**) PKA, and (**h**) ERK1, over 10 minutes. **i,j.** Quantification of γ-phosphate transferred from [^{32}P] ATP to the wild-type NHR-49 polypeptide by recombinant PKA over 10 minutes (**i**), or ERK1 over 5 minutes (**j**). n = 1 for both trials due to a failure to phosphorylate. **k,l.** (**k**)Transcriptomic and (**l**) proteomics analysis from whole worm extracts compares *kin-19* levels between empty vector control and *kin-19* RNAi conditions. Mean ± SEM. n = 4. Unpaired t-test (two-tailed) used for proteomics. Differential Expression Analysis in Two Groups with FDR correction (Qiagen CLC Workbench v9.5) used for transcriptomics. **m.** Relative abundance of *fat-7* transcripts determined by RNA-sequencing. Analysis compares Day 1 adults on an empty vector control and *kin-19* RNAi. Mean ± SEM. n = 4, statistics represent Differential Expression Analysis in Two Groups with FDR correction (Qiagen CLC Workbench v9.5). **n.** Gene Ontology analysis for molecular and cellular terms associated with differentially regulated genes when comparing worms on an empty vector control with *kin-19* RNAi via RNAseq.

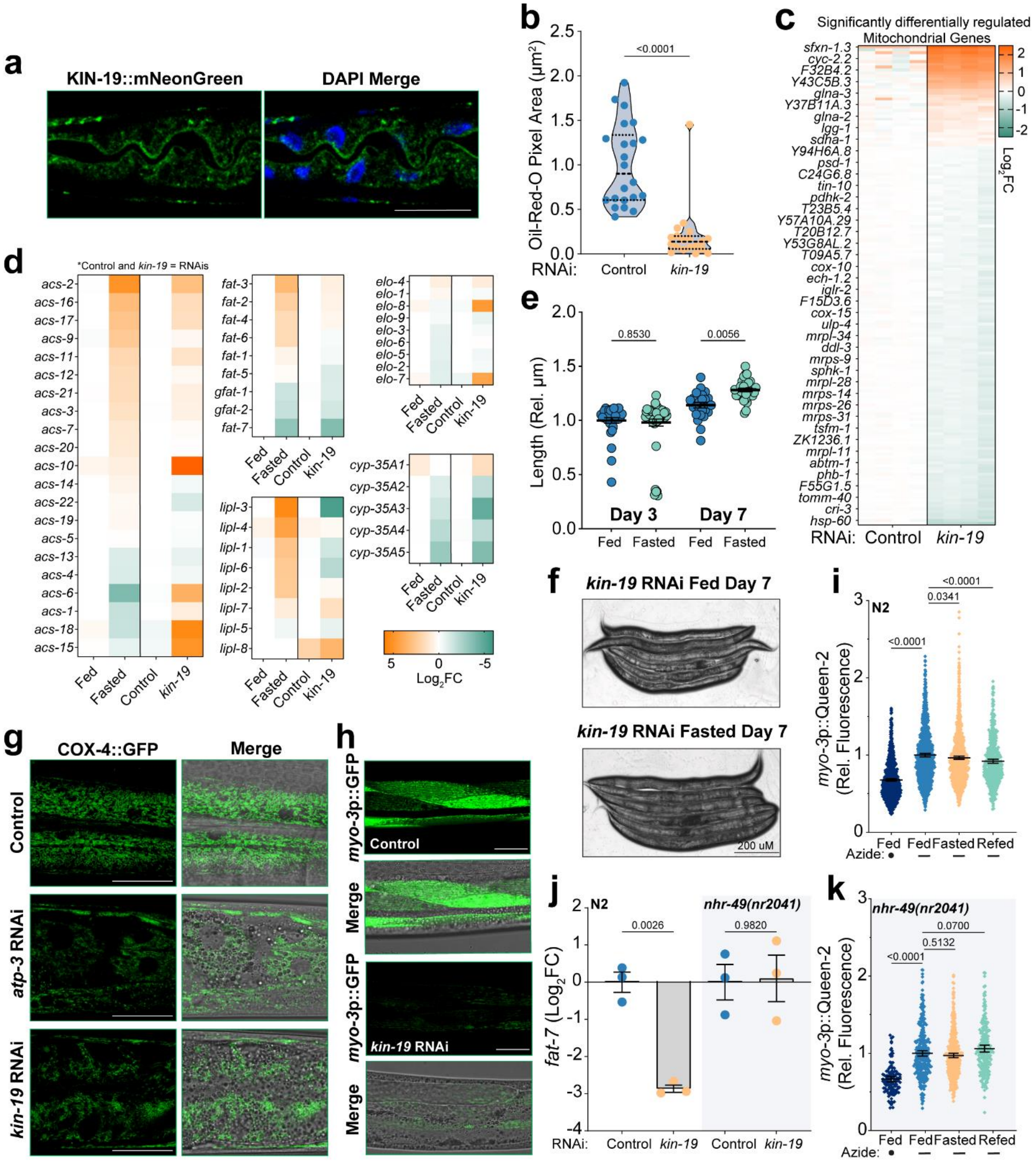

Supplementary figure 6: Impact of KIN-19 activity on the physiological changes associated with fasting

a. Fluorescence micrographs of *C. elegans* intestinal cells expressing the KIN-19::mNeonGreen2. Fluorescent tag is integrated at the endogenous locus. Scale bar = 25µm. Chromosomal DNA stained with DAPI. **b.** Quantification of Oil-Red-O staining comparing empty vector with *kin-19* RNAi treated worms. n = 22 and 19 over 3 biological trials from left to right, unpaired t-test (two-

tailed) used for statistics. **c.** Multiple heatmaps depict relative transcriptional changes in significantly differentially regulated genes associated with lipid metabolism such as ACSs, CYP35As, ELOs, FATs, and LIPLs. Analysis compares fed and fasted states to Day 1 adults under control RNAi to those under *kin-19* RNAi. **d.** Heatmap depicts relative transcriptional changes in significantly differentially regulated genes associated with mitochondria. Analysis compares Day 1 adults under control RNAi to those under *kin-19* RNAi. **e.** Quantification of body length for worms on *kin-19* RNAi determined through automated video surveillance (Wormtracker). All values are made relative to the fed Day 3 adult worms. Mean ± SEM. n = 33, 49, 25, and 30 from left to right, ordinary one-way ANOVA with Šídák's multiple comparisons test used for statistics. **f.** Micrographs of wild-type (N2) worms on *kin-19* RNAi at Day 7 of adulthood either fed or fasted at Day 1 of adulthood. Scale bar = 200µm. **g.** Fluorescence micrographs of Day 1 *C. elegans* intestinal cells expressing the COX-4::GFP treated with control, *kin-19,* or *atp-3* RNAi. Fluorescent tag is integrated at the endogenous locus. Scale bar = 25µm. **h.** Fluorescence micrographs of *C. elegans* body-wall muscle expressing the exogenously expressed *myo-3*p::GFP treated with control and *kin-19* RNAi. Scale bar = 25µm. **i.** Scatter plots from large-particle flow cytometry of transgenic worms on *kin-19* RNAi expressing the Queen-2m ATP sensor in body-wall muscle. Individual values are a ratio the fluorescence excitation at 405 nm to 488 nm with respective time-of-flight (TOF). Plots compare Day 1 adults under fed conditions to 24 hours of fasting and subsequent refeeding for 24 hours. Azide treatments serve as a negative control. Mean ± 95% CI. n = 1019, 1095, 1088 and 522 from left to right, ordinary one-way ANOVA with Šídák's multiple comparisons test used for statistics. **j.** Relative abundance of *fat-7* transcription determined by RT-qPCR. Analysis compares Day 1 adult wild-type or *nhr-49(nr2041)* mutant worms on empty vector control or *kin-19* RNAi. n = 3, ordinary one-way ANOVA with Šídák's multiple comparisons test used for statistics. **k.** Scatter plots from large-particle flow cytometry of *nhr-49(nr2041)* mutant animals on *kin-19* RNAi expressing the Queen-2m ATP sensor in body-wall muscle. Individual values are a ratio the fluorescence excitation at 405 nm to 488 nm with respective time-of-flight (TOF). Plots compare Day 1 adults under fed conditions to 24 hours of fasting and subsequent refeeding for 24 hours. Azide treatments serve as a negative control. Mean ± 95% CI. n = 116, 326, 440 and 211 from left to right, ordinary one-way ANOVA with Šídák's multiple comparisons test used for statistics.

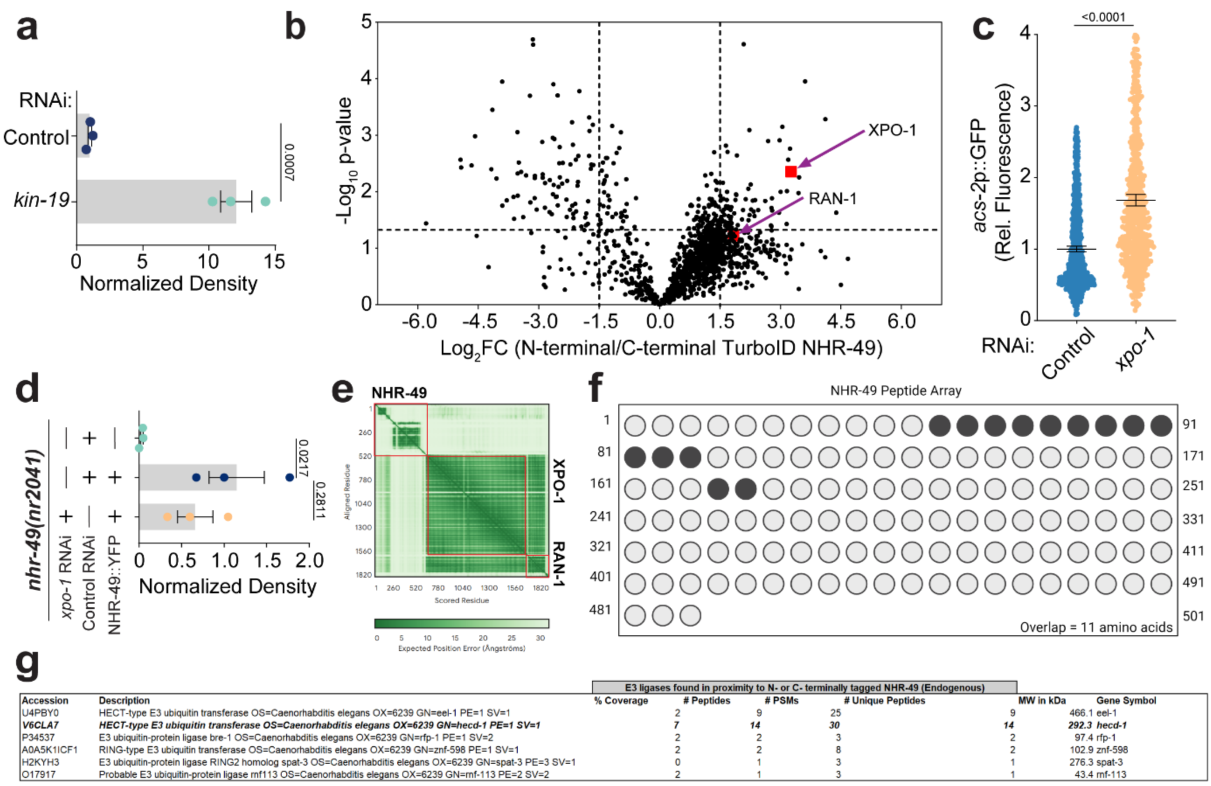

g

E3 ligases found in proximity to N- or C- terminally tagged NHR-49 (Endogenous)

Accession	Description	% Coverage	# Peptides	# PSMs	# Unique Peptides	MW in kDa	Gene Symbol
U4PBY0	HECT-type E3 ubiquitin transferase OS=Caenorhabditis elegans OX=6239 GN=eel-1 PE=1 SV=1	2	9	25	9	466.1	eel-1
V6CLA7	***HECT-type E3 ubiquitin transferase OS=Caenorhabditis elegans OX=6239 GN=hecd-1 PE=1 SV=1***	***7***	***14***	***30***	***14***	***292.3***	***hecd-1***
P34537	E3 ubiquitin-protein ligase bre-1 OS=Caenorhabditis elegans OX=6239 GN=rfp-1 PE=1 SV=2	2	2	3	2	97.4	rfp-1
A0A5K1ICF1	RING-type E3 ubiquitin transferase OS=Caenorhabditis elegans OX=6239 GN=znf-598 PE=1 SV=1	2	2	8	2	102.9	znf-598
H2KYH3	E3 ubiquitin-protein ligase RING2 homolog spat-3 OS=Caenorhabditis elegans OX=6239 GN=spat-3 PE=3 SV=1	0	1	3	1	276.3	spat-3
O17917	Probable E3 ubiquitin-protein ligase rnf113 OS=Caenorhabditis elegans OX=6239 GN=rnf-113 PE=2 SV=2	2	1	3	1	43.4	rnf-113

E3 ligases found in immunoprecipitated with NHR-49::GFP under fed and fasted conditions

Accession	Description	% Coverage	# Peptides	# PSMs	# Unique Peptides	MW in kDa	Gene Symbol
Q95XU3	***E3 ubiquitin-protein ligase OS=Caenorhabditis elegans OX=6239 GN=wwp-1 PE=1 SV=2***	***39***	***24***	***110***	***24***	***90.6***	***wwp-1***

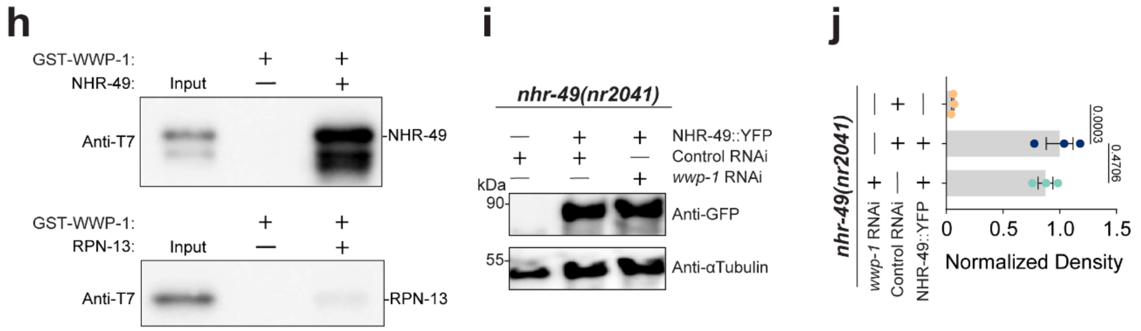

Supplementary figure 7: NHR-49 interactions with XPO-1 and WWP-1

a. Densitometry used to quantify abundances in NHR-49::YFP in *nhr-49(nr2041)* background showing band intensity under empty vector control and *kin-19* RNAi conditions. Mean ± SEM. n = 3. An unpaired t-test (two-tailed) was used for statistical analysis. See western blot Fig. 5b. **b.** Volcano plot of proteins enriched in proximity to the N- or C-terminally tagged NHR-49 TurboID. **c.** Scatter plots from large-particle flow cytometry of transgenic worms expressing *acs-2*p::GFP shows relative fluorescence per individual animal. Plots compare Day 1 adults on empty vector control or *xpo-1* RNAi. Mean ± 95% CI. n = 814 and 599 from left to right, an unpaired t-test (two tailed used for statistical analysis. **d.** Densitometry used to quantify abundances in NHR-49::YFP in *nhr-49(nr2041)* background measuring band intensity under empty vector control and *xpo-1* RNAi conditions. Mean ± SEM. n = 3. Ordinary one-way ANOVA with Dunnett's multiple

comparisons test used for statistics. See western blot Fig. 5e. **e.** Expected position error plot for interactions between NHR-49, XPO-1, and RAN-1 as determined by AlphaFold3. **f.** Schematic of NHR-49 polypeptide array used in Fig. 5g. Created in BioRender. Douglas, P. (2026) https://BioRender.com/a3wgh60 **g.** Filtering criteria of E3 ubiquitin ligases in proximity or that have immunoprecipitated with NHR-49. **h.** Western blot analysis of T7 epitope tagged NHR-49. Immunoprecipitations between NHR-49 and WWP-1 were performed using Glutathione S-Transferase (GST) epitope-tagged WWP-1 and subsequently immunoblotted for the T7 epitope on NHR-49. RPN-13 was included as negative control. **i.** Western blot of NHR-49::YFP in *nhr-49(nr2041)* background under empty vector control and *wwp-1* RNAi at Day 1 of adulthood. **j.** Densitometry used to quantify abundances in NHR-49::YFP in *nhr-49(nr2041)* background measuring band intensity under empty vector control and *wwp-1* RNAi conditions. Mean ± SEM. n = 3. Ordinary one-way ANOVA with Dunnett's multiple comparisons test used for statistics.

Supplementary table 1: *C. elegans* lifespan data

Figure	Strain, Treatment, RNAi	Avg. median lifespan	Observed/Total	% lifespan increase Fasted/Fed	p-value log-rank (Mendel-Cox)
1e	N2, fed, empty vector control RNAi	19	716/849		
1e	N2, fasted 24 hours, empty vector control RNAi	26.8	788/968	40.79	0.0139
1i	nhr-49 (nr2041), fed, empty vector control RNAi	11.2	853/1003		
1i	nhr-49 (nr2041), fasted 24 hours, empty vector control RNAi	17.6	721/901	57.14	0.0002
4j	N2, fed, *kin-19* RNAi	16.3	578/840		
4j	N2, fasted 24 hours, *kin-19* RNAi	17.7	492/548	8.16	0.1835
	nhr-49(n2041) , fed, *kin-19* RNAi	14.7	431/483		
	nhr-49(n2041) , fasted 24 hours, *kin-19* RNAi	14.7	416/454	0	>0.9999
Extended 3a	N2, fed, *atgl-1* RNAi	18	292/363		
Extended 3a	N2, fasted 24 hours, *atgl-1* RNAi	25.5	272/327	41.67	0.1257
Extended 3c	*nhr-49(nr2041)* , fed, *atgl-1* RNAi	12	136/157		
Extended 3c	*nhr-49(nr2041)* , fasted 24 hours, *atgl-1* RNAi	19	74/124	47.4	N/A
Extended 3b	N2, fed, *cpt-2* RNAi	17	210/281		
Extended 3b	N2, fasted 24 hours, *cpt-2* RNAi	24.5	260/338	44.12	0.278
Extended 4d	NHR-49::YFP in *nhr-49 (nr2041),* fed, empty vector control RNAi	17.5	103/144		
Extended 4d	NHR-49::YFP in *nhr-49 (nr2041),* fasted for 24 hours, empty vector control RNAi	23	58/76	31.43	N/A

Supplementary table 2: *In silico* predictions of NHR-49 and 19 kinases

Protein Name	iPTM	PTM
KGB-1	0.32	0.48
EGL-4	0.19	0.45
UNC-43	0.21	0.34
KIN-19	0.38	0.52
MVK-1	0.22	0.43
LET-754	0.29	0.45
KIN-2	0.23	0.41
SGK-1	0.19	0.37
PGK-1	0.15	0.46
PYK-1	0.13	0.54
CSNK-1	0.28	0.46
NDK-1	0.13	0.45
KIN-3	0.24	0.44
KIN-1	0.25	0.41
GSK-3	0.18	0.47
AIR-1	0.47	0.52
PAR-1	0.37	0.35
ARGK-1	0.18	0.45
MPK-1	0.29	0.49

Supplementary table 3: Kinase screening data

Šídák's post hoc test	Mean diff.	95.00% CI of diff.	Below threshold?	Summary	Adj. P Value	A-?	Kinase
Control vs. MVK-1	-0.00677	-0.08156 to 0.06802	No	ns	>0.9999	B	MVK-1
Control vs. NDK-1	-0.06743	-0.1458 to 0.01095	No	ns	0.1713	C	NDK-1
Control vs. GSK-3	-0.1082	-0.2075 to -0.008844	Yes	*	0.0204	D	GSK-3
Control vs. PAR-1	-0.1402	-0.2147 to -0.06573	Yes	****	<0.0001	E	PAR-1
Control vs. KGB-1	-0.1407	-0.2251 to -0.05635	Yes	****	<0.0001	F	KGB-1
Control vs. EGL-4	-0.1007	-0.1806 to -0.02088	Yes	**	0.0029	G	EGL-4
Control vs. UNC-43	-0.05783	-0.1466 to 0.03091	No	ns	0.6266	H	UNC-43
Control vs. KIN-19	-5.037	-5.111 to -4.962	Yes	****	<0.0001	I	KIN-19
Control vs. LET-754	-0.4402	-0.5314 to -0.3490	Yes	****	<0.0001	J	LET-754
Control vs. KIN-2	-1.291	-1.381 to -1.200	Yes	****	<0.0001	K	KIN-2
Control vs. SGK-1	-0.337	-0.4259 to -0.2481	Yes	****	<0.0001	L	SGK-1
Control vs. PGK-1	0.01493	-0.05951 to 0.08937	No	ns	>0.9999	M	PGK-1
Control vs. PYK-1	-0.1649	-0.2461 to -0.08374	Yes	****	<0.0001	N	PYK-1
Control vs. KIN-3	-0.07568	-0.1614 to 0.01003	No	ns	0.1426	O	KIN-3
Control vs. KIN-1	-0.04251	-0.2005 to 0.1155	No	ns	>0.9999	P	KIN-1
Control vs. MPK-1	-0.03451	-0.1121 to 0.04306	No	ns	0.9779	Q	MPK-1
Control vs. CSNK-1	-0.2291	-0.3184 to -0.1398	Yes	****	<0.0001	R	CSNK-1
Control vs. AIR-1	-0.1304	-0.2376 to -0.02331	Yes	**	0.0049	S	AIR-1
Control vs. ARGK-1	-0.1817	-0.2673 to -0.09609	Yes	****	<0.0001	T	ARGK-1

Supplementary table 4: Nucleocytoplasmic cofactor screen

Dunnett's post hoc test	Mean diff.	95.00% CI of diff.	Below threshold?	Summary	Adj. P Value	A-?	Protein
Control vs. EMR-1	-1.25E-08	-0.09028 to 0.09028	No	ns	>0.9999	B	EMR-1
Control vs. MEL-28	4.923E-08	-0.08695 to 0.08695	No	ns	>0.9999	C	MEL-28
Control vs. NPP-3	0.275	0.1466 to 0.4034	Yes	****	<0.0001	D	NPP-3
Control vs. NPP-6	0.1243	0.05586 to 0.1928	Yes	****	<0.0001	E	NPP-6
Control vs. NPP-8	0.5061	0.4217 to 0.5904	Yes	****	<0.0001	F	NPP-8
Control vs. NPP-7	0.1114	0.04447 to 0.1783	Yes	****	<0.0001	G	NPP-7
Control vs. NPP-4	0.04193	-0.03528 to 0.1191	No	ns	0.6672	H	NPP-4
Control vs. NPP-5	-0.09267	-0.1726 to -0.01278	Yes	*	0.0128	I	NPP-5
Control vs. NPP-18	-0.06127	-0.1402 to 0.01771	No	ns	0.2278	J	NPP-18
Control vs. NPP-23	-0.04536	-0.1340 to 0.04330	No	ns	0.7375	K	NPP-23
Control vs. NXT-1	-0.1708	-0.2547 to -0.08689	Yes	****	<0.0001	L	NXT-1
Control vs. THOC-2	-0.1778	-0.2617 to -0.09389	Yes	****	<0.0001	M	THOC-2
Control vs. THOC-5	0.03733	-0.04470 to 0.1194	No	ns	0.853	N	THOC-5
Control vs. THOC-7	-0.06422	-0.1532 to 0.02475	No	ns	0.3063	O	THOC-7
Control vs. BAF-1	0.1589	0.08701 to 0.2309	Yes	****	<0.0001	P	BAF-1
Control vs. HPL-1	0.03061	-0.04221 to 0.1034	No	ns	0.91	Q	HPL-1
Control vs. IMA-3	0.1837	0.1138 to 0.2537	Yes	****	<0.0001	R	IMA-3
Control vs. LMN-1	-0.01537	-0.08641 to 0.05566	No	ns	>0.9999	S	LMN-1
Control vs. NPP-12	0.09889	0.02741 to 0.1704	Yes	**	0.0013	T	NPP-12
Control vs. NPP-14	-0.06267	-0.1334 to 0.008054	No	ns	0.1144	U	NPP-14
Control vs. NPP-15	-0.06288	-0.1351 to 0.009359	No	ns	0.127	V	NPP-15
Control vs. NPP-16	-0.0621	-0.1357 to 0.01153	No	ns	0.1514	W	NPP-16
Control vs. NPP-19	-0.0117	-0.08350 to 0.06010	No	ns	>0.9999	X	NPP-19
Control vs. NPP-20	0.1866	0.1148 to 0.2584	Yes	****	<0.0001	Y	NPP-20
Control vs. NPP-24	-0.05156	-0.1230 to 0.01985	No	ns	0.3059	Z	NPP-24
Control vs. IMB-2	-0.0761	-0.1397 to -0.01250	Yes	**	0.009	AA	IMB-2
Control vs. IMB-3	0.3418	0.2774 to 0.4061	Yes	****	<0.0001	AB	IMB-3
Control vs. XPO-1	-0.2516	-0.3152 to -0.1880	Yes	****	<0.0001	AC	XPO-1
Control vs. XPO-2	0.1224	0.05892 to 0.1860	Yes	****	<0.0001	AD	XPO-2
