# Supplementary material for: Silencing lipid catabolism determines longevity in response to fasting
Source: Nat Commun. 2026 Jan 22;17:1919. doi: 10.1038/s41467-026-68764-y (PMC12923588; doi:10.1038/s41467-026-68764-y)
Supplement: Supplementary file 2 — Description Of Additional Supplementary File [file 41467_2026_68764_MOESM2_ESM.pdf]

Description of Additional supplementary files

- **Supplementary Data 1** – NHR-49: YFP IP mass spec data.
- **Supplementary Data 2** – Oligonucleotides and primers list.
- **Supplementary Data 3** – Primary and secondary antibodies list.
