# Supplementary material for: Silencing lipid catabolism determines longevity in response to fasting
Source: Nat Commun. 2026 Jan 22;17:1919. doi: 10.1038/s41467-026-68764-y (PMC12923588; doi:10.1038/s41467-026-68764-y)
Supplement: Supplementary file 7 — Reporting summary [file 41467_2026_68764_MOESM7_ESM.pdf]

nature portfolio

Corresponding author(s): Peter M. Douglas

Last updated by author(s): Jan 7, 2026

Reporting Summary

Nature Portfolio wishes to improve the reproducibility of the work that we publish. This form provides structure for consistency and transparency in reporting. For further information on Nature Portfolio policies, see our Editorial Policies and the Editorial Policy Checklist.

Statistics

For all statistical analyses, confirm that the following items are present in the figure legend, table legend, main text, or Methods section.

n/a	Confirmed	
☐	☒	The exact sample size (*n*) for each experimental group/condition, given as a discrete number and unit of measurement
☐	☒	A statement on whether measurements were taken from distinct samples or whether the same sample was measured repeatedly
☐	☒	The statistical test(s) used AND whether they are one- or two-sided<br>*Only common tests should be described solely by name; describe more complex techniques in the Methods section.*
☐	☒	A description of all covariates tested
☐	☒	A description of any assumptions or corrections, such as tests of normality and adjustment for multiple comparisons
☐	☒	A full description of the statistical parameters including central tendency (e.g. means) or other basic estimates (e.g. regression coefficient) AND variation (e.g. standard deviation) or associated estimates of uncertainty (e.g. confidence intervals)
☐	☒	For null hypothesis testing, the test statistic (e.g. *F*, *t*, *r*) with confidence intervals, effect sizes, degrees of freedom and *P* value noted<br>*Give P values as exact values whenever suitable.*
☒	☐	For Bayesian analysis, information on the choice of priors and Markov chain Monte Carlo settings
☒	☐	For hierarchical and complex designs, identification of the appropriate level for tests and full reporting of outcomes
☒	☐	Estimates of effect sizes (e.g. Cohen's *d*, Pearson's *r*), indicating how they were calculated

Our web collection on statistics for biologists contains articles on many of the points above.

Software and code

Policy information about availability of computer code

Data collection	Software used for data collection include FlowPilot (v1.6.18, Union Biometrica), Leica Application Suite X (v3.5.5, LAS X), Image Lab (v6.1, Bio-Rad), WormLab (2024.01.01 64-bit), ZEN Blue Software (v. 2.3), Proteome Discoverer (v3.0), AutoDock-GPU (2021–present), Google DeepMind's AlphaFold2, Google DeepMind's AlphaFold3.
Data analysis	Software used for data analysis include Bio-Rad CFX manager (v3.1), MATLAB (R2023b), Excel (v16, Microsoft), Prism (v10.2.3 ,GraphPad), CLC Genomics (v23, CLC Bio).

For manuscripts utilizing custom algorithms or software that are central to the research but not yet described in published literature, software must be made available to editors and reviewers. We strongly encourage code deposition in a community repository (e.g. GitHub). See the Nature Portfolio guidelines for submitting code & software for further information.

Data

Policy information about availability of data

All manuscripts must include a data availability statement. This statement should provide the following information, where applicable:

- Accession codes, unique identifiers, or web links for publicly available datasets
- A description of any restrictions on data availability
- For clinical datasets or third party data, please ensure that the statement adheres to our policy

All transcriptomic datasets generated during this study will be deposited in the NCBI Gene Expression Omnibus (GEO) upon request and/or acceptance.

Research involving human participants, their data, or biological material

Policy information about studies with human participants or human data. See also policy information about sex, gender (identity/presentation), and sexual orientation and race, ethnicity and racism.

Reporting on sex and gender	This study did not involve any human participant, data, or material
Reporting on race, ethnicity, or other socially relevant groupings	This study did not involve any human participant, data, or material
Population characteristics	This study did not involve any human participant, data, or material
Recruitment	This study did not involve any human participant, data, or material
Ethics oversight	This study did not involve any human participant, data, or material

Note that full information on the approval of the study protocol must also be provided in the manuscript.

Field-specific reporting

Please select the one below that is the best fit for your research. If you are not sure, read the appropriate sections before making your selection.

☒ Life sciences ☐ Behavioural & social sciences ☐ Ecological, evolutionary & environmental sciences

For a reference copy of the document with all sections, see nature.com/documents/nr-reporting-summary-flat.pdf

Life sciences study design

All studies must disclose on these points even when the disclosure is negative.

Sample size	When sample size calculation was relevant to perform, desired power was set to 0.80 and alpha to 0.05. Sample size calculation was performed utilizing standard equations for either dichotomous or continuous variables, as appropriate. Large particle flow cytometry experiments: sampling a smaller portion of the larger animal populations was not necessary because large particle flow cytometry enabled rapid collection and analysis for the entire population of worms. qPCR: Desired at minimum to detect a 2-fold change. Preliminary experiments revealed that the coefficient of variation (CV) for gene expression data was 0.15. With the above power and significance, we obtained a minimum sample size of approximately 3. Microscopy: To evaluate dhs-3 intensity, initial experiments revealed starved displayed 71% intensity loss. To detect a difference of at least 20% with power of 0.8, a minimum of 10 worms are needed per group. To evaluate oil-red-o intensity, preeliminary experiments revealed kin-19 RNAi displayed 82% intensity loss. To detect a difference of at least 20% with power of 0.8, a minimum of 18 worms are needed per group. When sample sizes were not predetermined prior to experimentation, all experiments were repeated to produce a minimum sample size of n=3.
Data exclusions	Data exclusions only applied for large worm populations. Outliers were removed using the ROUT method (Q=1%) prior to analysis.
Replication	To ensure reproducibility and consistency, appropriate internal positive and negative controls were included when applicable, allowing for effect size standardization across experiments. Furthermore, each experiment was conducted a minimum of three times to validate the reliability of the results. In the case of the peptide array in Fig. 5g, PepStar peptide microarrays through JPT are highly standardized and internally controlled. The immobilized peptide array included 15mer peptides (overlapping each other by 11 residues) spanning the long isoform C of the NHR-49 protein. Through this design, all core amino acid sequences within the NHR-49 protein are represented in at least 3 independent peptide spots and conclusions drawn from the array are supported by in silico binding data presented in the manuscript.
Randomization	At the beginning of each experiment, samples were pooled and then randomly assigned to either the control or experimental conditions.
Blinding	While blinding was not necessary for large sample sizes, a rigorous blinding procedure was implemented for nucleocytoplasmic distribution analysis. Plates were assigned condition-specific numbers, randomly shuffled, and relabeled with a neutral word. Independent researchers then selected ten worms per condition, generating an average to compare with the first author's data. Data were retained if the two measurements aligned within 10%, ensuring objective and reproducible quantification.

Reporting for specific materials, systems and methods

We require information from authors about some types of materials, experimental systems and methods used in many studies. Here, indicate whether each material, system or method listed is relevant to your study. If you are not sure if a list item applies to your research, read the appropriate section before selecting a response.

Materials & experimental systems

n/a	Involved in the study
☐	☒ Antibodies
☒	☐ Eukaryotic cell lines
☒	☐ Palaeontology and archaeology
☐	☒ Animals and other organisms
☒	☐ Clinical data
☒	☐ Dual use research of concern
☒	☐ Plants

Methods

n/a	Involved in the study
☒	☐ ChIP-seq
☐	☒ Flow cytometry
☒	☐ MRI-based neuroimaging

Antibodies

Antibodies used	polyclonal anti-GFP (1:7500, Rabbit; Cat. #A6455, Invitrogen), monoclonal anti-TUBA4A (1:5000, Mouse; Cat. #T6074, Sigma), IgG IRDye 680RD (1:15000, Goat, Cat. #926-68070, LICORbio), polyclonal anti-Rabbit IgG IRDye 800CW (1:15000, Goat, Cat. #926-32211, LICORbio), monoclonal anti-XPO1 antibody (1:1000, Mouse, Santa Cruz, Cat. #sc-74454), monoclonal anti-T7 primary antibody (1:5000, Mouse, Cat. #69522 MilliporeSigma), polyclonal HRP-conjugated anti-mouse secondary antibody (1:7500; Goat, Cat. #A4416, MilliporeSigma)
Validation	Western blot antibodies were validated by confirming the expected molecular weight, reported by the manufacturer, via SDS-PAGE. Antibodies have been utilized in prior publications by our laboratory and/or our collaborator's lab.

Animals and other research organisms

Policy information about studies involving animals; ARRIVE guidelines recommended for reporting animal research, and Sex and Gender in Research

Laboratory animals	C elegans: N2: Bristol (wild-type), AGP33a: nhr-49(nr2041); glmEx8[nhr-49p::NHR-49::GFP; myo-2p::mCherry], LIU1: ldrIs1[dhs-3p::DHS-3::GFP; unc-76(+)], STE68: nhr-49(nr2041), JJ2586: cox-4(zu476[COX-4::eGFP::3xFLAG]) I, WBM170: wbmEx57[acs-2p::GFP + rol-6(su1006)], and SJ4103: zcIs14[myo-3::GFP(mit)]; GA2001: wuIs305[myo-3p::Queen-2m]; PMD166: utsIs4[nhr-49p::NHR-49::GFP; myo-3p::mCherry]; nhr-49(nr2041), PMD142: ldrIs1[dhs-3p::DHS-3::GFP; unc-76(+)]; nhr-49(nr2041), PMD319: nhr-49(syb10204[nhr-49::3xHA::TurboID]), PMD320: nhr-49c(syb10203[3xHA::TurboID::nhr-49c]), and PMD124: utsIs3[rab-11.2p::YFP::unc-54 3'UTR]. The following strains were generated for this study: PMD192: utsEx24[pept-1p::YFP::unc-54 3' UTR], PMD261: utsIs10[pept-1p::NHR-49::YFP::unc-54 3'UTR], nhr-49(nr2041), PMD262: utsIs11[pept-1p::NHR-49(Δ295-422)::YFP::unc-54 3'UTR]; nhr-49(nr2041), PMD263: utsIs15[pept-1p::NHR-49(S114A)::YFP::unc-54 3' UTR]; nhr-49(nr2041), PMD314: utIs13[pept-1p::NHR-49(s114d)::YFP::unc54 3'UTR]; nhr-49(nr2041), PD17: kin-19(syb8063[kin-19::mNeonGreen2]), and PMD311: wuIs305[myo-3p::Queen-2m]; nhr-49(nr2041).
Wild animals	no wild animals were used for this study.
Reporting on sex	Sex was not considered in this study.
Field-collected samples	no field-collected sampless were used for this study.
Ethics oversight	Not applicable.

Note that full information on the approval of the study protocol must also be provided in the manuscript.

Plants

Seed stocks	no plants were used in this study.
Novel plant genotypes	no plants were used in this study.
Authentication	no plants were used in this study.

Flow Cytometry

Plots

Confirm that:

☐ The axis labels state the marker and fluorochrome used (e.g. CD4-FITC).

☒ The axis scales are clearly visible. Include numbers along axes only for bottom left plot of group (a 'group' is an analysis of identical markers).

☐ All plots are contour plots with outliers or pseudocolor plots.

☒ A numerical value for number of cells or percentage (with statistics) is provided.

Methodology

Sample preparation	Worms were washed off 100 mm plates with M9 and rinsed three times with M9 before 100 µl worm pellets were loaded into a 96-well plate for acquisition. To prevent cross-well contamination, M9 was loaded into wells between samples.
Instrument	COPAS FP-250 with LPS (union biometrica)
Software	Flowpilot (v 1.6.18)
Cell population abundance	This is not an applicable issue for C. elegans large particle flow cytometry as they are easily identified by size and optical density
Gating strategy	C. elegans were separated from bacterial food source and debris based on time of flight and extinction. C. elegans that did not pass through the flow cell in an straightened fashion were identified by an abnormal extinction peak height to integral extinction ratio and were gated out of the final population. The resulting cleaned C. elegans population was then analyzed for fluorescence. Gating was reporting in the extended data figures.

☒ Tick this box to confirm that a figure exemplifying the gating strategy is provided in the Supplementary Information.
